# Overcoming phagocytosis resistance of hypervirulent *Klebsiella pneumoniae* by directly targeting capsules

**DOI:** 10.15698/mic2026.02.870

**Published:** 2026-02-16

**Authors:** Shogo Tsubaki, Touya Toyomoto, Rika Tanaka, Jin Imai, Juntaro Matsuzaki, Katsuto Hozumi, Hitoshi Tsugawa

**Affiliations:** 1 Transkingdom Signaling Research Unit, Division of Host Defense Mechanism, Tokai University School of Medicine; Isehara, Kanagawa, 259-1193, Japan; 2 Department of Immunology, Division of Host Defense Mechanism, Tokai University School of Medicine; Isehara, Kanagawa, 259-1193, Japan; 3 Department of Ophthalmology, Keio University School of Medicine; Shinjuku-ku, Tokyo, 160-8582, Japan; 4 Department of Clinical Health Science, Tokai University School of Medicine; Isehara, Kanagawa, 259-1193, Japan; 5 Institute of Medical Sciences, Tokai University; Isehara, Kanagawa, 259-1193, Japan; 6 Division of Interdisciplinary Genetics and Nanomedicine, Research Center for Drug Discovery, Keio University Faculty of Pharmacy; Minato-ku, Tokyo, 105-8512, Japan; 7 Human Biology-Microbiome-Quantum Research Center (WPI-Bio2Q), Keio University; Shinjuku-ku, Tokyo, 160-8582, Japan

**Keywords:** capsule synthesis, small RNA, phagocytosis, hypermucoviscosity assay, RmpA, Fur

## Abstract

Capsular polysaccharides (CPS) are key virulence factors in *Klebsiella pneumoniae* and are closely associated with the K1 and K2 hypervirulent serotypes. Herein, we demonstrate that introducing nonspecific RNA (sgRNA) into *K. pneumoniae* ATCC43816 (Kp-pET-sgRNA), a K2 serotype strain classified as hypervirulent (hvKp), results in marked capsule loss and reduced hypermucoviscosity. Capsule loss and reduced hypermucoviscosity in Kp-pET-sgRNA were confirmed by comparison with the wild-type strain (Kp-WT) using transmission electron microscopy, hypermucoviscosity assays, and string tests. Mechanistically, we found that overexpression of sgRNA by introducing the pET-sgRNA plasmid led to gene deletion in the *rmpADC* operon, a key virulence determinant located on mobile chromosomal elements. Additionally, the mRNA expression of *manC*, which are chromosomal *cps*-related genes, was significantly repressed. In contrast, introduction of pET-sgRNA did not alter the mRNA expression of *galF* or *wzi*. The results revealed that capsule loss and reduced hypermucoviscosity in Kp-pET-sgRNA resulted from synergistic downregulation of both the *rmpADC* operon and *manC*. Loss of capsule synthesis and reduction of hypermucoviscosity in *K. pneumoniae* caused by sgRNA overexpression significantly decreased resistance to phagocytosis by macrophages but did not influence susceptibility to meropenem or colistin. The findings reveal an unexpected consequence of plasmid-mediated sgRNA introduction, where overexpression of sgRNA abolishes phagocytic resistance by disrupting capsule biosynthesis and reducing hypermucoviscosity in *K. pneumoniae*. This study highlights a promising strategy for disarming hypervirulent *K. pneumoniae* by directly targeting its key virulence factors and provides novel insights into antibacterial therapeutic approaches against this clinically significant pathogen.

## INTRODUCTION

*Klebsiella pneumoniae* is a Gram-negative rod bacterium and one of the commensal bacteria in the human gastrointestinal tract. It is known to readily cause liver abscesses and pneumonia in older adults and immunocompromised patients. Furthermore, in recent years, carbapenem resistance in *K. pneumoniae* has surged dramatically, particularly in Europe and the United States, leading to severe systemic infections that pose a direct threat to life 1.

*K. pneumoniae* is recognized as a pathogen with high rates of multidrug resistance (MDR) 2, 3. The World Health Organization (WHO) has defined the six most important multidrug-resistant bacteria commonly found in hospitals (ESKAPE), and the “K” in ESKAPE refers to *K. pneumoniae* 4. The US CDC has also identified these bacteria, including *K. pneumoniae*, as a serious public health threat 5.

*K. pneumoniae* is broadly classified into classical (cKp) and hypervirulent (hvKp) strains 6–9. cKp causes hospital-acquired infections, hvKp causes community-acquired infections 10, 11. Notably, cKp typically affects immunocompromised individuals, particularly older adults, whereas hvKp can cause severe infections including liver abscesses and pneumonia, even in healthy adults. The reported intestinal colonization rates for cKp and hvKp range from 18.8–87.7% and 0–16.7%, respectively 12. Notably, isolates from fecal samples of healthy carriers have been reported to share serotypes and genotypes with hvKp strains 13, and hvKp was recovered from the fecal samples of two healthy relatives of a patient with a liver abscess, suggesting possible human-to-human transmission via the fecal–oral route 14. In addition to the epidemiological observations, we recently demonstrated that intestinal mucosal macrophages play a pivotal role in restricting the translocation of intestinally colonized cKp or hvKp to the liver, lungs, and mesenteric lymph nodes in murine *in vivo* infection models 15, 16. The findings suggested that the gut is an important reservoir for not only cKp but also hvKp, which breaches the intestinal barrier and translocates directly to extraintestinal organs, including the liver, causing systemic infections such as liver abscesses.

The genetic background of hvKp is highly diverse, with sequence type (ST) 23 being the most prevalent lineage 17, 18. In early 2024, the WHO requested data collection to understand the global state of drug resistance, prompted by the increasing detection of hvKp ST23 strains harboring carbapenemase genes conferring resistance to carbapenem. As hvKp ST23 strains can infect both healthy and immunocompromised individuals and exhibit a strong propensity for invasive diseases, the WHO recommends the progressive enhancement of laboratory diagnostic capacity to ensure early and accurate identification of hvKp.

*K. pneumoniae* is classified into at least 79 capsular serotypes, including eight associated with hypervirulence: K1, K2, K5, K16, K20, K54, K57, and KN1, with K1 and K2 being the most prevalent 19, 20. The K1 serotype is strongly linked to hvKp ST23 21. Geographic variation exists, with K1 predominating in Asia and K2 more common in Europe and North America 22, 23. The K2 strain of *K. pneumoniae* is highly virulent, owing to its strong phagocytic resistance in mice. All 26 strains of the K2 serotype isolates collected from liver abscess patients in three different regions of Asia have been reported contain a regulator of the mucoid phenotype gene (*rmpA*), which is deeply involved in the regulation of capsule production 24.

*K. pneumoniae* lacks specific virulence factors; however, its polysaccharide capsule, which envelops the outer membrane, is the major determinant of its pathogenicity. Capsule production contributes to the hypermucoviscous phenotype by enabling resistance to phagocytosis, complement-mediated killing, antimicrobial peptides and antibody-dependent clearance 25–30. The capsule of *K. pneumoniae* contributes to resistance to phagocytosis by macrophages, and non-encapsulated *K. pneumoniae* strains are easily phagocytosed by macrophages 31, 32. Moreover, heterogeneity in phagocytic sensitivity has been observed in clinical respiratory isolates of *K. pneumoniae* 33. Geest *et al*. classified 19 clinical respiratory isolates into macrophage phagocytosis-susceptible and phagocytosis-resistant strains and found that phagocytosis-susceptible strains exhibited significantly reduced capsule formation and mucoviscosity compared with phagocytosis-resistant strains 33. The findings suggested that, in human infections with *K. pneumoniae*, loss of capsule formation significantly reduces the immune resistance of the bacterium, leading to reduced pathogenicity.

Capsule biosynthesis genes are organized within the capsular polysaccharide synthesis (cps) locus, which spans from *galF* to *ugd* and comprises approximately 20 genes encoding transport/export proteins and glycosyltransferases essential for capsule assembly. The abovementioned genes are regulated by three promoters upstream of *galF*, *wzi*, and *manC* 34, 35. The serotype-specific *wzi* gene encodes an outer membrane protein that anchors CPSs; deletion of *wzi* results in an acapsular phenotype 36–38. Additionally, high capsule productivity and the hypermucoviscosity phenotype are driven by the *rmpADC* operon, a key virulence determinant located on the large virulence plasmid (pLVPK) and mobile chromosomal elements 39. RmpA and RmpC function as transcriptional regulators with DNA-binding domains, whereas RmpD interacts with capsule export machinery to confer a hypermucoviscous phenotype 40–42. Experimental evidence from murine pneumonia models has demonstrated that deletion of the *rmpADC* gene markedly reduces bacterial colonization in the lungs 40, suggesting that targeting the *rmpADC* operon could abolish hvKp pathogenicity. Consequently, the negative regulation of *rmpADC* gene expression represents a promising strategy for preventing infection, as this regulation may attenuate hvKp virulence or promote its conversion to the cKp phenotype.

Understanding the molecular mechanisms underlying the interaction between *K. pneumoniae* and host cells is essential for elucidating its pathogenesis. Our research group previously investigated the pathogenesis of *K. pneumoniae*, focusing on its interactions with host cells including macrophages 15, 16. In previous studies, we constructed CRISPR sensor cells to monitor transkingdom RNA transfer. The abovementioned sensor cells were designed as LacZ reporter cells that express Cas9 endonuclease from *Streptococcus pyogenes* (Sp-Cas9) and contain a specific sgRNA target site that regulates LacZ expression. To test this sensor system, a single-guide RNA (sgRNA) was designed with a sequence intended only to target the sensor construct within the reporter cells; the sgRNA did not target any specific endogenous genes in either the host or bacteria. The sgRNA was ligated into two expression plasmids, pET30b and pGFP, and subsequently introduced into *K. pneumoniae* ATCC43816 (K2 serotype, hypervirulent hvKp strain). The K2 serotype is highly virulent, largely due to its strong phagocytic resistance and the presence of virulence genes such as *rmpA*, which regulate capsule production 24. Interestingly, we observed an unexpected phenotypic change; *K. pneumoniae* transformed with the sgRNA-expressing pET30 plasmid exhibited a distinct phenotype characterized by capsule loss and reduced hypermucoviscosity. Expressing the same sgRNA using the pGFP plasmid did not cause this effect. The present study was designed to elucidate the underlying molecular and physiological mechanisms responsible for the specific phenotypic changes resulting from pET30-mediated sgRNA expression and to clarify its effect on the bacterial characteristics of *K. pneumoniae*.

Recent studies have shown that bacterial small RNAs (sRNAs) inhibit transcriptional activity by base pairing with target mRNAs, thereby modulating protein expression and activity 43. Additionally, some sRNAs interact with mRNA coding sequences (CDSs) to promote RNase E-dependent mRNA decay, rather than simply blocking translation 44. The regulation of gene expression by sRNAs also plays a central role in bacterial carbon metabolism, highlighting their importance as key intracellular regulatory elements 45. In this study, we found that the mRNA expression of *rmpA*, *rmpD*, and *rmpC* was completely inhibited in the Kp-pET-sgRNA strains, demonstrating that overexpressed synthetic sgRNAs can target the *rmpADC* operon and neutralize hvKp pathogenicity. Our findings provide novel insights into preventive strategies against hvKp by directly inhibiting capsule formation through the targeted disruption of the *rmpADC* operon.

## RESULTS

### Loss of capsule synthesis in *K. pneumoniae* following the overexpression of specific RNA sequence, sgRNA

**Figure 1:**
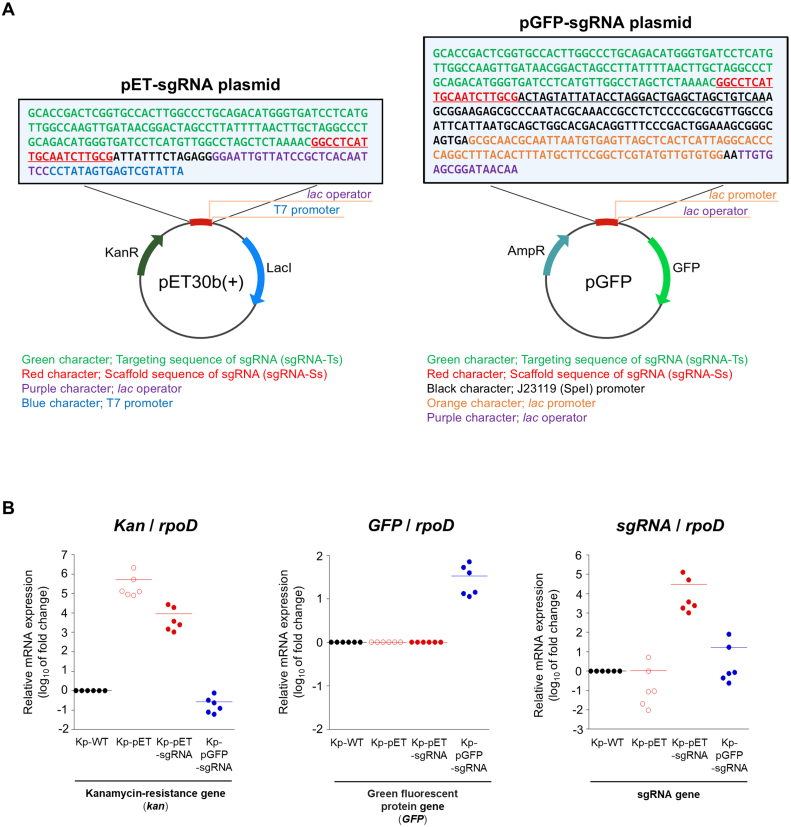
Construction of sgRNA-overexpressing *K. pneumoniae*. **(A)** Vector maps showing insertion of the sgRNA sequence along with T7 and *lac* promoter sequences into the pET30b and pGFP plasmids. The sgRNA sequence consists of the targeting sequence (sgRNA-Ts), indicated in green, and the scaffold sequence (sgRNA-Ss), indicated in red, inserted into the multiple cloning site (MCS). The *lac* operator is shown in purple, the T7 promoter in blue, and the *lac* promoter in orange. **(B)** Quantitative real-time RT-PCR analysis. The endogenous control used *rpoD*. Fold changes in gene expression were calculated using the 2^ − Δ Δ CT method, with the expression level of each gene in Kp-WT as the reference. Based on these values, the relative expression levels of each gene between strains are shown as fold differences in the graph.

The sgRNA sequence developed in a previous study consists of a target sequence (sgRNA-Ts) intended to bind to our custom Cas9-based sensor construct, along with a scaffold sequence (sgRNA-Ss) (Figure 1**A**). This sgRNA sequence was cloned into two distinct expression vectors for subsequent transformation into *K. pneumoniae* ATCC43816 (Kp-WT): first, into the pET30b plasmid, where its expression is driven by the T7 promoter, and then into the pGFP plasmid, where its expression is controlled by the lac promoter. The resulting recombinant plasmids pET30b-sgRNA and pGFP-sgRNA were individually introduced into *K. pneumoniae* by electroporation to generate the experimental strains used in this study.

Using electroporation, we constructed Kp-pET strains by transfection with the pET plasmid only, Kp-pET-sgRNA strains by transfection with pET-sgRNA, and Kp-pGFP-sgRNA strains by transfection with pGFP-sgRNA. Quantitative real-time RT-PCR was performed to confirm sgRNA expression in the Kp-pET-sgRNA and Kp-pGFP-sgRNA strains. As shown in Figure 1**B**, we compared the mRNA expression levels of the kanamycin resistance gene (*kan*), green fluorescent protein gene (*GFP*), and sgRNA in each strain using Kp-WT as the reference. The mRNA expression of *Kan* was markedly high in Kp-pET plasmid-transfected Kp-WT strains, whereas *GFP* mRNA expression was detected only in Kp-pGFP-sgRNA strains (Figure 1**B**). The findings confirmed successful plasmid introduction into Kp-WT strains. Although sgRNA mRNA expression was observed in both the Kp-pET-sgRNA and Kp-pGFP-sgRNA strains, the expression level of Kp-pET-sgRNA was approximately 1800-fold higher than that of Kp-pGFP-sgRNA (Figure 1**B**).

**Figure 2:**
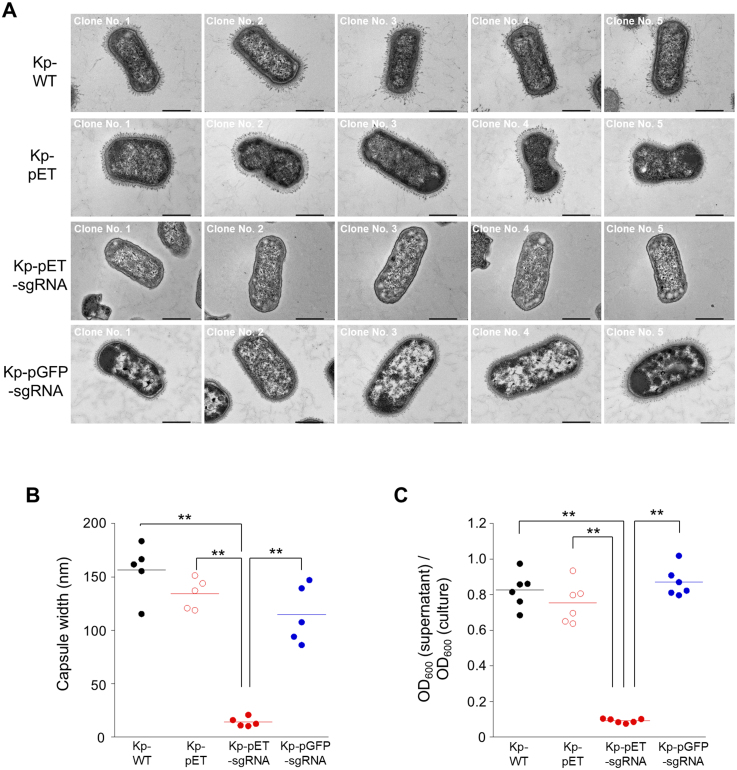
Loss of capsular polysaccharide and reduced hypermucoviscosity in *K. pneumoniae* following pET-sgRNA introduction. **(A)** TEM analysis of Kp-WT, Kp-pET-sgRNA, and Kp-pGFP-sgRNA cultured in DMEM. Scale bar is 500 nm. **(B)** The thickness of the capsule is measured from the TEM images using Image J analysis software. Sample were compared using the Tukey’s range test, with **p < 0.01. **(C)** Analysis of mucus viscosity loss by hypermucoviscosity assay. To evaluate hypermucoidy, Kp-WT, Kp-pET, and Kp-pET-sgRNA strains were cultured until late stationary phase, followed by centrifugation at 1,000 × g for 5min. The optical density at 600 nm (OD ${}_{600}$ ) of the supernatant was determined and expressed as a fraction of the initial OD ${}_{600}$ . Sample were compared using the Tukey’s range test, with **p < 0.01.

To examine whether the introduction of sgRNA into Kp-WT induces structural changes in the bacterial cell, we cultured Kp-WT, Kp-pET (pET plasmid-introduced Kp-WT), Kp-pET-sgRNA (pET-sgRNA-introduced Kp-WT), and Kp-pGFP-sgRNA (pGFP-sgRNA-introduced Kp-WT) strains in Dulbecco’s modified Eagle’s medium (DMEM) and examined their morphology using transmission electron microscopy (TEM) (Figure 2**A**). TEM analysis revealed a complete loss of the capsule structure in all Kp-pET-sgRNA clones (Figure 2**A**). We then measured the capsule thickness of individual bacterial cells by TEM using the ImageJ software. Capsule thickness in Kp-pET-sgRNA was significantly reduced compared with that in Kp-WT, Kp-pET, and Kp-pGFP-sgRNA (Figure 2**B**). Additionally, the hypermucoviscosity assay demonstrated a significant reduction in hypermucoviscosity in Kp-pET-sgRNA compared with that in other strains (Figure 2**C**). Moreover, the String test yielded positive results for the Kp-WT strain (Supplemental Movies S1), whereas the Kp-pET-sgRNA strain tested negative (Supplemental Movies S2). The results indicated that Kp-pET-sgRNA exhibited a marked decrease in both capsule integrity and hypermucoviscosity relative to the other strains.

### Overexpression of sgRNA via the pET30b plasmid directly inhibits *rmpADC* operon expression

**Figure 3:**
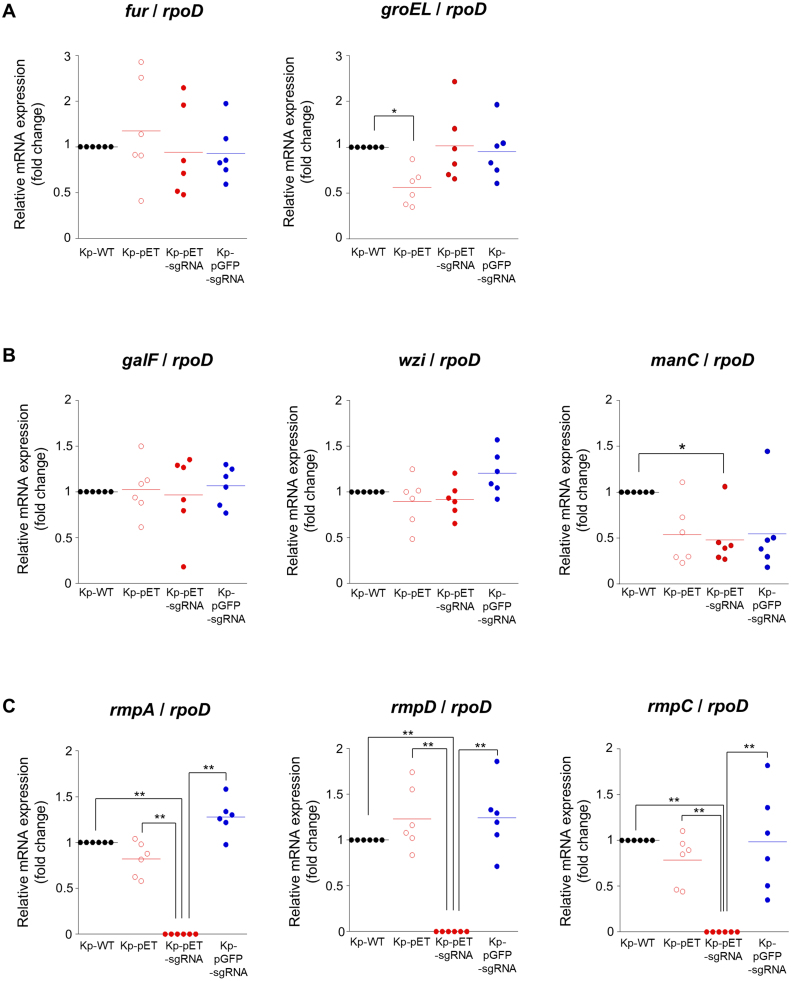
Quantitative real-time RT-PCR analysis in sgRNA-overexpressing *K. pneumoniae*. Quantitative real-time RT-PCR analysis of gene expression in Kp-WT, Kp-pET, Kp-pET-sgRNA, and Kp-pGFP-sgRNA: fur and groEL **(A)**; galF, wzi, and manC **(B)**; rmpA, rmpD, and rmpC **(C)**. The endogenous control used *rpoD*. Fold changes in gene expression were calculated using the 2^ − Δ Δ CT method, with the expression level of each gene in Kp-WT as the reference. Based on these values, the relative expression levels of each gene between strains are shown as fold differences in the graph. Sample were compared using the Tukey’s range test, with *p < 0.01, **p < 0.01.

We next investigated the mechanism underlying the loss of capsule synthesis and reduced hypermucoviscosity in the Kp-pET-sgRNA. *K. pneumoniae* encodes 20 genes involved in cps on its chromosome 35 (Supplementary Figure S1A). The *cps* locus consists of three operons, represented by *galF*, *wzi*, and *manC* (Supplementary Figure S1A). In addition to the *cps* locus, the *K. pneumoniae* chromosome also encodes the *rmpADC* operon, which is composed of *rmpA*, *rmpC*, and *rmpD*, which are involved in hypermucoviscosity (Supplementary Figure S1B). The mRNA expression of *rmpA* is transcriptionally regulated by the transcription factor ferric uptake regulator (Fur) 41. Fur is an intracellular Fe ${{}^{2}}^{+}$ -responsive transcription factor that also negatively regulates the mRNA expression of cps-related genes on the chromosome 46. Moreover, *rmpA*, which functions as a transcriptional regulator of *manC*, is negatively regulated by Fur 47–49. We first examined whether introduction of pET-sgRNA altered *fur* mRNA expression by quantitative real-time RT-PCR. As shown in Figure 3**A**, there are no significant differences in *fur* expression levels observed among Kp-WT, Kp-pET, Kp-pET-sgRNA, Kp-pGFP-sgRNA. The results suggest that the introduction of pET-sgRNA does not directly affect Fur-mediated regulation of *cps*-related genes expression. Reactive oxygen species (ROS), including H ${}_{2}$ O ${}_{2}$ , are also known to promote the upregulation of *cps*-related genes through a decrease in intracellular cAMP levels 4, 5. The bacterial heat shock protein GroEL, whose expression is elevated under osmotic stress and H ${}_{2}$ O ${}_{2}$ exposure, is widely used as an indicator of bacterial stress responses. The results of quantitative RT-PCR analysis revealed that the mRNA expression of *groEL* in Kp-pET-sgRNA was comparable to that in Kp-WT, Kp-pET, and Kp-pGFP-SgRNA (Figure 3**A**). The expression level of *groEL* in Kp-pET was significantly reduced compared to Kp-WT, but this decrease in *groEL* expression was not associated with the loss of capsule synthesis or the reduction in hypermucoviscosity (Figure 3**A**). These results indicate that the introduction of pET-sgRNA does not act as a stress-inducing factor associated with the loss of capsule synthesis or the reduction of hypermucoviscosity in *K. pneumoniae*.

Next, to examine whether introduction of pET-sgRNA contributes to the directly silencing of cps genes, we assessed the mRNA expression of *galF*, *wzi*, and *manC*, a gene located within the chromosomal cps locus 48. The mRNA expression levels of *galF* and *wzi* showed no significant differences among Kp-WT, Kp-pET, Kp-pET-sgRNA, and Kp-pGFP-sgRNA, suggesting that these genes are not involved in the observed loss of capsule structure and reduction of hypermucoviscosity in Kp-pET-sgRNA (Figure 3**B**). In contrast, *manC* expression was decreased in all plasmid-transformed strains compared with Kp-WT, and notably, Kp-pET-sgRNA exhibited a significant reduction in *manC* expression relative to Kp-WT (Figure 3**B**). Because the downregulation of *manC* was detected in all plasmid-transformed strains (Kp-pET, Kp-pET-sgRNA, and Kp-pGFP-sgRNA) and showed no significant differences among these strains, decreased *manC* expression was thought to be the not only factor inducing capsule loss and reducing the hypermucoviscosity in Kp-pET-sgRNA. Therefore, we further analyzed the expression of *rmpA*, *rmpC*, and *rmpD*. The results revealed that the mRNA expression of *rmpA*, *rmpC*, and *rmpD* was undetectable in Kp-pET-sgRNA and significantly lower than in Kp-WT, Kp-pET, and Kp-pGFP-sgRNA (Figure 3**C**). These findings suggest that capsule loss and reduced hypermucoviscosity in Kp-pET-sgRNA result from the synergistic down-regulation of both the *rmpADC* operon and *manC*.

### *rmpADC* operon deletion characterizes the Kp-pET-sgRNA with abolished capsule synthesis and decreased hypermucoviscosity

**Figure 4:**
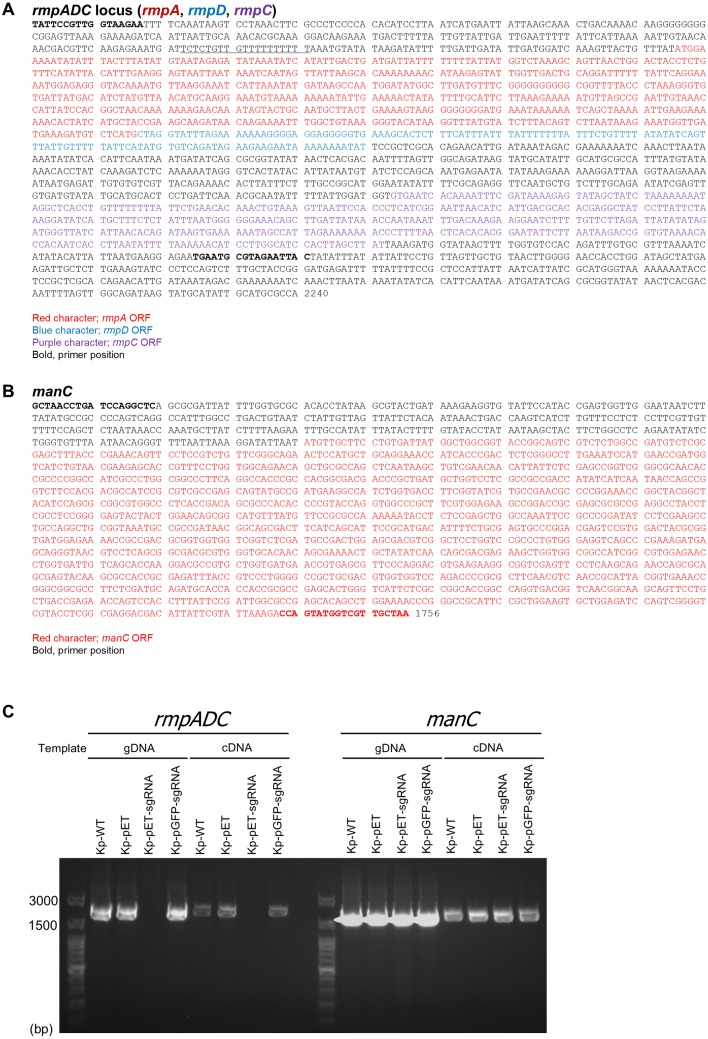
Deletion of the *rmpADC* operon from the *K. pneumoniae* chromosome following sgRNA overexpression via introduction of the pET-sgRNA plasmid. Nucleotide sequence of the *rmpADC* operon **(A)** and *manC* ORF **(B)** obtained from the genome of *K. pneumoniae* ATCC43816. **(A)** The *rmpA* ORF is shown in red, the *rmpD* in blue, and the *rmpC* in purple. **(B)** The *manC* ORF is shown in red. Primer sequence used for PCR amplification is shown in bold. **(C)**Agarose gel electrophoresis of PCR products for *rmpADC* and *manC* in each strain.

Since RT-qPCR analysis did not detect any mRNA expression of *rmpA*, *rmpD*, or *rmpC* in Kp-pET-sgRNA (Figure 3**C**), we next assessed whether cDNA of the *rmpADC* operon had been synthesized. PCR primers were designed based on the *rmpADC* operon ORF sequence obtained from the genome information of the *K. pneumoniae* ATCC 43816 strain (Figure 4**A,**shown in bold). Additionally, PCR primers for *manC* (Figure 4**B,**shown in bold), whose expression was found to be reduced in RT-qPCR analysis, were also designed. Using these primers, PCR was performed for the *rmpADC* operon and *manC* with genomic DNA and cDNA from each strain as templates. PCR products for the *rmpADC* operon were not detected in Kp-pET-sgRNA, regardless of whether genomic DNA or cDNA was used as the template but were detected in the other strains (Figure 4**C**). This indicates that the absence of *rmpADC* mRNA expression in Kp-pET-sgRNA is attributable to deletion of the *rmpADC* gene. On the other hand, PCR products for *manC* were detected in Kp-pET-sgRNA, as in other strains, suggesting that the reduced *manC* mRNA expression is likely attributable to deletion of the transcriptional regulator *rmpA* gene (Figure 4**C**). To examine the cause of the *rmpADC* gene deletion, we investigated the nucleotide sequence approximately 1,600 bp upstream and 1,000 bp downstream of the *rmpADC* locus. Several base substitutions were observed; however, no evidence of gene replacement or homologous recombination was detected in the sequenced region (Supplementary Figure S2). These findings suggest that *rmpADC* gene deletion caused by overexpressing sgRNA in Kp-pET-sgRNA leads to the loss of capsule synthesis and a decrease in hypermucoviscosity.

### Loss of capsule synthesis and reduced hypermucoviscosity caused by pET30b-mediated sgRNA overexpression abolished phagocytosis resistance in *K. pneumoniae*

**Figure 5:**
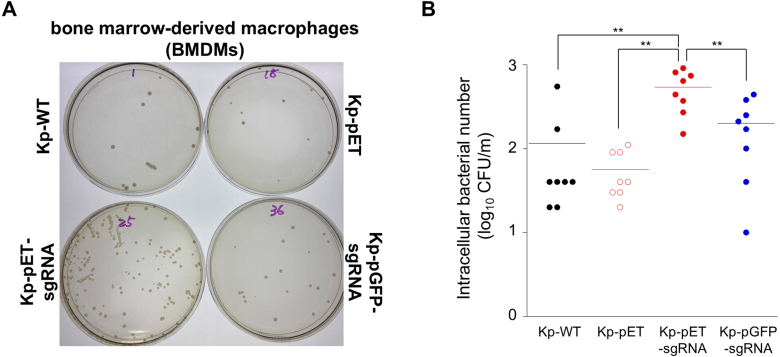
K. pneumoniae, which loses its capsule due to the introduction of pET-sgRNA, becomes more susceptible to phagocytosis. Measurement of phagocytic activity against *K. pneumoniae* by BMDMs using gentamicin killing assay. BMDMs were infected with the bacteria at an MOI of 1 (2 × 10 ${}^{5}$ bacteria/well) for 2 h. After gentamicin treatment (4 h) to kill extracellular bacteria, the cells were lysed with 1% Triton X-100, and lysates were plated on LB agar. Bacterial numbers were counted after 24 h of cultivation at 37 ${}^{\circ }$ C. Sample were compared using the Tukey’s range test, with **p < 0.01.

Capsule of *K. pneumoniae* contributes to resistance to phagocytosis by macrophages, and non-encapsulated *K. pneumoniae* strains are known to be easily phagocytosed by macrophages 50. To determine whether inhibition of capsule synthesis and reduced hypermucoviscosity of *K. pneumoniae* by introduction of pET-sgRNA also abolishes macrophage phagocytosis resistance, we infected murine bone-marrow derived macrophages (BMDMs) with each *K. pneumoniae* strain at a multiplicity of infection (MOI) of 1 for 120 min, and then the intracellular bacterial number were counted. In BMDMs, the number of intracellular bacteria in the Kp-pET-sgRNA strains was significantly higher than that in the Kp-WT, Kp-pET, or Kp-pGFP-sgRNA strains, demonstrating that these Kp-pET-sgRNA strains were more easily phagocytosed by macrophages (Figure 5). The results show that the capsular loss and reduced hypermucoviscosity-inducing activity of pET-sgRNA upon transfection of *K. pneumoniae* aids the macrophage bacterial clearance response of this bacterium and may be applicable to the development of novel prevention and treatment methods for this bacterial infection.

### Loss of capsule synthesis and reduced hypermucoviscosity caused by pET30b-mediated sgRNA overexpression do not alter antibiotic susceptibility of *K. pneumoniae*

It has been suggested that hvKP may acquire antibiotic resistance through multiple mechanisms 51, and capsule synthesis and hypermucoviscosity have also been identified as bacteriological characteristics of hvKp that contribute to drug resistance 52. Then to determine whether capsule loss induced by pET-sgRNA transformation affects antibiotic susceptibility, we measured the minimum inhibitory concentrations (MICs) of four representative antibiotics: gentamicin (aminoglycoside), meropenem (carbapenem), colistin (polypeptide with membrane-disrupting activity), and levofloxacin (fluoroquinolone). The MIC values for these antibiotics scarcely differed among Kp-WT, Kp-pET, Kp-pET-sgRNA, and Kp-pGFP-sgRNA (**Table 1**). These findings indicate that capsule loss and reduced hypermucoviscosity caused by the deletion of *rmpADC* operon does not significantly influence susceptibility to these antibiotics in *K. pneumoniae*.

## DISCUSSION

The bacterial capsule, a polymeric polysaccharide located on the bacterial surface, plays a pivotal role in immune evasion by inhibiting phagocytosis and complement activation 53. Additionally, it contributes to biofilm formation, host cell adhesion, and invasion 54, 55. In pathogens such as *Streptococcus pneumoniae* and *Neisseria meningitidis*, capsule synthesis is indispensable for the establishment of infection and is needed to cause an infectious disease 56, 57. The present study demonstrated that introducing a specific RNA sequence via the pET30b plasmid effectively disrupts capsule synthesis and reduces hypermucoviscosity in *K. pneumoniae*, thereby compromising its resistance to phagocytosis and attenuating its ability to evade host immune responses. Our findings highlight the potential of plasmid-mediated gene modifications to induce unanticipated phenotypic changes in bacteria. Moreover, they emphasize the importance of rigorous phenotypic validation following forced expression of exogenous sequences. Notably, our findings offer novel insights into the therapeutic strategies targeting capsule synthesis and hypermucoviscosity in *K. pneumoniae*, a pathogen of increasing clinical concern owing to its multidrug resistance and virulence.

Among the two-component regulatory systems (TCS) of *K. pneumoniae*, a system that positively regulates capsular synthesis is known 58. RcsAB is a TCS that induces capsular synthesis by positively regulating the transcription of *galf*. RcsAB responds to the iron concentration and cooperates with *fur* to regulate *galf* expression 59. In addition, KvgAS, a TCS system consisting of the sensor histidine kinase KvgS and the response regulator KvgA, senses oxidative stress, external iron, bile, and osmotic stress, and is involved in regulating capsular synthesis 58. In particular, iron-limited environments or NaCl stress decreases *kvgAS* expression, leading to reduced capsular synthesis 60. In the present study, we observed no changes in the mRNA expression of *groEL* or *fur* in Kp-pET-sgRNA, which abolished capsular synthesis (Figure 3**A**). This result suggested that sgRNA overexpression in bacteria does not induce intracellular or extracellular stress that is detectable by capsular synthesis-related TCS systems that downregulate capsular synthesis.

Bacterial sRNAs bind to specific target mRNAs within CDSs and promote RNase E-dependent mRNA decay 43, 44. Notably, Goh *et al*. demonstrated that the OmrB sRNA of *K. pneumoniae* within the CDS region of the capsule synthesis transcription factor *kvrA* markedly suppressed KvrA expression, thereby inhibiting capsule formation 61. In the present study, the overexpression of Kp-pET-sgRNA, which resulted in loss of capsule formation, significantly reduced *rmpADC* operon mRNA expression (Figure 3**C**). This observation led us to hypothesize that the introduced sgRNA base pairs in a specific region of the *rmpADC* operon induced mRNA degradation. However, PCR using genomic DNA from Kp-pET-sgRNA failed to amplify *rmpADC* operon sequences, suggesting that the introduction of pET-sgRNA into Kp-WT resulted in deletion of the *rmpADC* operon (Figure 4**C**). The findings indicated that the introduced sgRNA eliminated the *rmpADC* operon through a mechanism distinct from that of conventional sgRNA-mediated mRNA decay, resulting in loss of capsule formation and reduced hypermucoviscosity.

As shown in Figure 4**C**, although PCR was performed to amplify *rmpADC* from the Kp-pET-sgRNA genomic DNA, no PCR product was detected, suggesting that the *rmpADC* region was deleted by sgRNA overexpression in Kp-pET-sgRNA. Our findings indicated that *rmpADC* gene deletion in Kp-pET-sgRNA results in loss of capsule synthesis and decreased hypermucoviscosity. DNA sequencing analysis of the upstream and downstream regions of the *rmpADC* deletion region did not detect large-scale insertions or recombinations of foreign genes (Supplementary Figure S2). Additionally, no extensive regions of homology to the *rmpADC* gene within the pET-sgRNA plasmid were present, and PCR using genomic DNA as a template did not detect any amplification products, including PCR products of different sizes (Figure 4**C**). In the present study, we were unable to determine the mechanism by which sgRNA overexpression induces gene deletion in the *rmpADC* operon of Kp-pET-sgRNA. Further analyses, including a whole-genome analysis of Kp-pET-sgRNAs, are required to clarify this point. Deletion of the *rmpADC* operon in Kp-pET-sgRNA is presumed to result from DNA double-strand breaks (DSBs) induced by sgRNA overexpression. Although non-coding small RNAs are known to be involved in DSB repair in eukaryotic cells 62, 63, non-coding small RNAs capable of excising and removing targeted DNA have been reported in ciliates, a unicellular eukaryote 64–67. Although further studies are needed to clarify whether sgRNA overexpression in *K. pneumoniae* is associated with DSBs targeting the *rmpADC* operon, our results demonstrated that sgRNA overexpression induced by the introduction of pET-sgRNA induced gene deletion of the *rmpADC* operon in genomic DNA.

The K1 and K2 serotypes of *K. pneumoniae* are widely recognized as markers of hvKp and other highly pathogenic strains. In contrast, Geest *et al*. reported that clinical isolates from patients with respiratory diseases included the K2 serotype strain, which is highly susceptible to phagocytosis and has low pathogenicity 33. This finding indicated that the K2 serotype does not necessarily correlate with the phagocytosis-resistant phenotype in a clinical setting. In addition, Yu *et al*. demonstrated that strains harboring the *rmpA* gene and exhibiting a hypermucoviscous phenotype are associated with *in vivo* pathogenicity, regardless of the K1/K2 serotype 68. The findings suggested that the clinical virulence of hvKp is primarily driven by the acquisition of phagocytic resistance through enhanced capsule synthesis and hypermucoviscosity. Therefore, caution is warranted when defining the virulence of *K. pneumoniae* based solely on the K1/K2 serotype. The present study showed that introduction of pET-sgRNA into *K. pneumoniae* strains exhibiting increased capsule synthesis and hypermucoviscosity resulted in abolishing these traits, thereby attenuating macrophage phagocytosis resistance. To determine whether targeting capsule synthesis and mucoviscosity alone is sufficient to reduce the virulence of all clinical isolates, further investigation into the relationship between phagocytic resistance and other bacterial components, including lipopolysaccharides, outer membrane proteins, and fimbriae, is required. In addition, whether pET-sgRNAs exert similar biological effects across diverse clinical isolates requires further evaluation using a larger strain set. Nonetheless, our findings highlight a potential technical approach for neutralizing the key virulence factors of *K. pneumoniae* and enhancing macrophage-mediated clearance.

Capsule formation has been reported to protect *K. pneumoniae* cells from the antimicrobial activities of two antimicrobial peptides (AMPs), polymyxin B and colistin 69, 70. However, other studies have shown that non-encapsulated strains exhibit higher MIC values for polymyxin B than encapsulated strains 71. Thus, the contribution of the capsule to the AMP susceptibility of *K. pneumoniae* remains unclear. Similarly, regarding virulence, particularly intestinal colonization, studies using capsule-deficient mutants have produced conflicting conclusions regarding the role of the capsule 35. The findings suggested that numerous genes involved in capsule synthesis do not uniformly contribute to a single phenotype. Therefore, when analyzing the role of the capsule in drug susceptibility and virulence, the phenotypic effect of the targeted gene must be carefully evaluated and gene selection for knockout experiments should be deliberate. In the Kp-pET-sgRNA strain, in which the *rmpADC* operon was deleted via sgRNA overexpression, the colistin MIC remained unchanged, but the phagocytic resistance of macrophages was significantly reduced. TEM analysis confirmed the complete loss of capsule formation in Kp-pET-sgRNA; however, *rmpADC* did not directly mediate capsule synthesis and was implicated in hypermucoviscosity. Our results indicated that the *rmpADC* operon is not a determinant of drug resistance in *K. pneumoniae* but plays a critical role in conferring phagocytosis resistance.

Our findings indicated that the introduction of our specific exogenous plasmid, pET-sgRNA, can target and delete the *rmpADC* operon, thereby abolishing capsule synthesis, which defines the hypervirulence of *K. pneumoniae*. Specifically, our results were as follows: (1) Introduction of *K. pneumoniae* with a pET30b plasmid carrying an overexpression of sgRNA within the bacterial cells led to loss of capsule synthesis and a reduction in hypermucoviscosity. (2) In *K. pneumoniae* strains in which capsule synthesis was abolished and hypermucoviscosity was reduced owing to sgRNA overexpression via the pET30b plasmid, *rmpADC* was deleted. (3) Loss of capsule synthesis and reduction in hypermucoviscosity associated with *rmpADC* deletion markedly decreased resistance to macrophage phagocytosis. (4) Loss of capsule synthesis due to *rmpADC* deletion does not affect antibiotic susceptibility. The findings indicated that the *rmpADC* gene, which defines hypervirulence in *K. pneumoniae* 40, 72, 73, is a deletion target through the constructed pET30b-sgRNA plasmid. Consequently, transformation with the pET30b-sgRNA plasmid may potentially induce a reduction in *K. pneumoniae* virulence, representing a valuable insight for developing preventive measures against hypervirulent strains.

## MATERIALS AND METHODS

### Bacterial cultures

*K. pneumoniae* ATCC43816 was grown overnight at 37 ${}^{\circ }$ C on Luria–Bertani (LB) agar plates (Nacalai Tesque Inc., Kyoto, Japan; cat. no. 20067–85). Bacterial density was estimated by measuring the optical density of cultures at 600 nm. For the mRNA expression analysis of capsular synthesis genes, *K. pneumoniae* cultured in Dulbecco’s Modified Eagle Medium (DMEM) (Gibco, Waltham, MA, USA; cat. no. 11965092) for 15 hours was collected and used.

### Cell culture

Murine BMDMs were prepared by flushing bone marrow from femurs and tibiae with DMEM and were cultured in in Dulbecco’s modified Eagle’s medium (DMEM; Gibco, cat. no. 11965092) supplemented with 10% FBS and 20 ng/mL recombinant mouse macrophage-colony stimulating factor (PeproTech, Rocky Hill, NJ, USA, cat. no. 315–02) for 7 days.

### Construction of *K. pneumoniae* strains expressing sgRNA

A non-specific single-guide RNA sequence was designed to consist of a target sequence (sgRNA-Ts, GCACCGACTCGGTGCCACTTGGCCCTGCAGACATGGGTGATCCTCATGTTGGCCAAGTTGATAACGGACTAGCCTTATTTTAACTTGCTAGGCCCTGCAGACATGGGTGATCCTCATGTTGGCCTAGCTCTAAAAC) targeting a repressor gene responsive to the Sp-Cas9-based CRISPR/Cas9 system and a scaffold sequence (sgRNA-Ss, GGCCTCATTGCAATCTTGCG). The sgRNA was ligated into pET30b plasmid or pGFP plasmid using the NEBuilder HiFi DNA Assembly (New England Biolabs, Ipswich, MA, USA, cat no. E2621L) so that it was under transcriptional control of the T7 promoter or lac promoter, respectively. *K. pneumoniae* ATCC43816 strains were washed three times with 10% glycerol on ice, and electroporated with the pET30b plasmid carrying a single-guide RNA insert (pET-sgRNA) or pGFP plasmid carrying a single-guide RNA insert (pGFP-sgRNA) using a MicroPulser (Bio-Rad, Hercules, CA, USA).

### Transmission Electron Microscopic (TEM) analysis

The bacteria were fixed by resuspending them in a TEM sample buffer containing 1% glutaraldehyde overnight at 4 ${}^{\circ }$ C. The samples were pipetted onto Formvar-coated 200-mesh nickel grids (Ted Pella Inc., Redding, CA, USA) and allowed to settle for 25 min. After the grids were air-dried, TEM images were obtained using a JEM-1400 transmission electron microscope (JEOL, Tokyo, Japan) at 100 kV.

### Hypermucoviscosity assay

Each *K. pneumoniae* strain was cultured overnight in 5 ml of DMEM at 37 ${}^{\circ }$ C. Cells were pelleted by centrifugation at 1,000 × g for 5 min at room temperature. The optical density at 600 nm (OD ${}_{600}$ ) of the upper 1000 μ L of the supernatant was measured using a spectrophotometer. Data were expressed as the ratio of the OD ${}_{600}$ of the supernatant to that of the original culture.

### Antibiotic susceptibility testing

The minimum inhibitory concentration (MIC) was determined using the serial two-fold dilution method. Each *K. pneumoniae* strain was cultured overnight in LB medium and subsequently diluted 1,000 with fresh LB medium. The antibiotics used were gentamicin (aminoglycoside, Fujifilm Wako Pure Chemical Co. Ltd, Osaka, Japan, cat no. 079-02973), meropenem (carbapenem, Sigma-Aldrich, St. Louis, MO, USA, cat no. M2574), colistin (polypeptide with membrane-disrupting activity, Fujifilm Wako Pure Chemical Co. Ltd, cat no. 032-20941.), and levofloxacin (fluoroquinolone, Fujifilm Wako Pure Chemical Co. Ltd, cat no. 125-05941). The bacterial suspensions were incubated with serially diluted each antibiotic drug for 16 hours at 37°C;. Bacterial growth was assessed by measuring the optical density (OD) at 595 nm.

### Quantitative RT-PCR

Total RNA was extracted from *K. pneumoniae*, using the SV Total RNA Isolation System (Promega, Madison, WI, USA; cat. no. Z3100). Complementary DNA was synthesized with the PrimeScript RT Reagent Kit (TaKaRa, Ohtsu, Japan; cat. no. RR037A). Quantitative PCR was conducted on QuantoStudio 3 (Thermo Fisher Scientific, Waltham, MA, USA) employing PowerTrack SYBR Green Master Mix (Thermo Fisher Scientific, cat. no. A46012). The following primers were used: *Kan* mRNA, forward 5 ${}^{\prime }$ -GATAATGTCGGGCAATCAGG and reverse 5 ${}^{\prime }$ -AGTACGGATAAAATGCTTGATGG; *GFP* mRNA, forward 5 ${}^{\prime }$ -TTTCACTGGAGTTGTCCCAA and reverse 5 ${}^{\prime }$ -GAAAAGCATTGAACACCATA; sgRNA mRNA, forward 5 ${}^{\prime }$ -GTTTTAGAGCTAGGCCAACAT and reverse 5 ${}^{\prime }$ -CCTCATGTTGGCCAAGT; *rpoD* mRNA, forward 5 ${}^{\prime }$ -GATGCTGTTGTCGTCATCGC and reverse 5 ${}^{\prime }$ -CTGTCCGATCTGATCACCGG; *fur* mRNA, forward 5 ${}^{\prime }$ -CGGCATCGTGACTCGTCATA and reverse 5 ${}^{\prime }$ -CGCACAGTGACCGTACAGAT; *groEL* mRNA 74, forward 5 ${}^{\prime }$ -AAGACACCACCACCATCATC and reverse 5 ${}^{\prime }$ -TCGCTTCTTCGATCTGCTTAC; *galF* mRNA, forward 5 ${}^{\prime }$ -ATGATCGCCCGCTTTAACGA and reverse 5 ${}^{\prime }$ -GCCATCAGATCGGAGTCCAG; *wzi* mRNA, forward 5 ${}^{\prime }$ -GTCGACCGCAATCATTCAGC and reverse 5 ${}^{\prime }$ -CTCACCAACCATCTGCCCAT; *manC* mRNA, forward 5 ${}^{\prime }$ -CGTTCCCAGGACGTGAAGAA and reverse 5 ${}^{\prime }$ -AATGCGGTTGACGTTGAAGC; *rmpA* mRNA, forward 5 ${}^{\prime }$ -AGAGTATTGGTTGACTGCAGGA and reverse 5 ${}^{\prime }$ -TTTAGGGTAAAACCGCCCCC; *rmpD* mRNA, forward 5 ${}^{\prime }$ -TGGCTGTAAAGGGTACATAAGGT and reverse 5 ${}^{\prime }$ -AAGAGTGCTTTCACCCCCTC; *rmpC* mRNA, forward 5 ${}^{\prime }$ -CGCACACGAGGCTATCCTTA and reverse 5 ${}^{\prime }$ -TCCGTGTGTGAGTTAAAAGGGT.

### DNA sequencing

The 5’-upstream and 3’-downstream regions of the *rmpADC* operon were amplified by PCR using the following primers: the 5’-upstream region of the *rmpADC* operon, forward 5’-TATCTTACACCACTCACAT and reverse 5’-TTCTTACCAACGGAATA; the 3’-downstream region of the *rmpADC* operon, forward 5’-TGAATGCGTAGAATTAC and reverse 5’-GGAAACGACATAAGGAC. The nucleotide sequences of each PCR product were directly sequenced by using the ABI 3500XL genetic analyzer (Applied Biosystems, Foster City, CA, USA) in accordance with the protocol of the BigDye Terminator v3.1 Cycle Sequencing Kit method (Thermo Fisher Scientific, cat. no. 4336919).

### *In vitro K. pneumoniae* infection

BMDMs cells (2 × 10 ${}^{5}$ cells/well) were seeded into 12-well plates (IWAKI, Shizuoka, Japan; cat. no. 3815012). *K. pneumoniae* strains were precultured overnight in LB broth. Cells were infected with bacteria at a multiplicity of infection (MOI) of 1 (2 × 10 ${}^{5}$ bacteria per well) for 2 h, followed by incubation with DMEM with gentamicin (Nacalai Tesque, cat. no. 11980-14) for 4 h to eliminate extracellular bacteria. Subsequently, cells were lysed in PBS supplemented with 1% Triton X-100, and the lysates were plated onto LB agar. Colony-forming units (CFUs) were enumerated after 24 h of incubation at 37 ${}^{\circ }$ C.

## SUPPLEMENTAL MATERIAL

All supplemental data for this article are available online at http://microbialcell.com/researcharticles/2026a-tsubaki-microbial-cell/.

.

.

## CONFLICT OF INTEREST

H. Tsugawa received funding for joint research from Taiyo Kagaku Co. Ltd. and Tsumura Corporation. Funding agencies played no role in the study of design, data collection and analysis, decision to publish, or manuscript preparation.

## ABBREVIATIONS

AMP – antimicrobial peptide

BMDMs – bone-marrow derived macrophages

CDS – coding sequence

DSB – DNA double-strand break

CPS – capsular polysaccharides

hvKp – hypervirulent

MIC – minimum inhibitory concentrations

MOI – multiplicity of infection

sgRNA – single-guide RNA

TCS – two-component regulatory system

TEM – transmission electron microscopy

WHO – World Health Organization

WT – wildtype

cKp – classical *Klebsiella pneumoniae*
