# Supplementary material for: Overcoming phagocytosis resistance of hypervirulent Klebsiella pneumoniae by directly targeting capsules
Source: Microb Cell. 2026 Feb 16;13:103–16. doi: 10.15698/mic2026.02.870 (PMC12925632; doi:10.15698/mic2026.02.870)
Supplement: Supplementary file 1 — . [file mic-13-103-s01.pdf]

Supplemental Material

Overcoming phagocytosis resistance of hypervirulent Klebsiella pneumoniae by directly targeting capsules

Shogo Tsubaki[1], Touya Toyomoto[1], Rika Tanaka[2,3], Jin Imai[4,5], Juntaro Matsuzaki[6,7], Katsuto Hozumi[2], Hitoshi Tsugawa[1,5]*

[1]Transkingdom Signaling Research Unit, Division of Host Defense Mechanism, Tokai University School of Medicine; Isehara, Kanagawa, 259-1193, Japan
[2]Department of Immunology, Division of Host Defense Mechanism, Tokai University School of Medicine; Isehara, Kanagawa, 259-1193, Japan
[3]Department of Ophthalmology, Keio University School of Medicine; Shinjuku-ku, Tokyo, 160-8582, Japan.
[4]Department of Clinical Health Science, Tokai University School of Medicine; Isehara, Kanagawa, 259-1193, Japan
[5]Institute of Medical Sciences, Tokai University; Isehara, Kanagawa, 259-1193, Japan
[6]Division of Interdisciplinary Genetics and Nanomedicine, Research Center for Drug Discovery, Keio University Faculty of Pharmacy; Minato-ku, Tokyo, 105-8512, Japan
[7]Human Biology-Microbiome-Quantum Research Center (WPI-Bio2Q), Keio University; Shinjuku-ku, Tokyo, 160-8582, Japan

***Corresponding author:**

Hitoshi Tsugawa, Ph.D.

Transkingdom Signaling Research Unit, Division of Host Defense Mechanism, Tokai University School of Medicine

143 Shimokasuya, Isehara, Kanagawa 259-1193, Japan

TEL: +81-463-93-1121

E-mail: tsugawa.hitoshi.r@tokai.ac.jp

Supplementary Figure S1

A

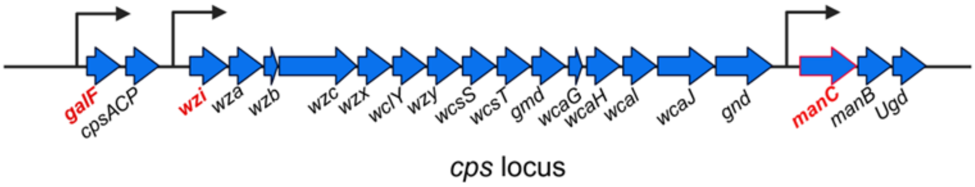

B

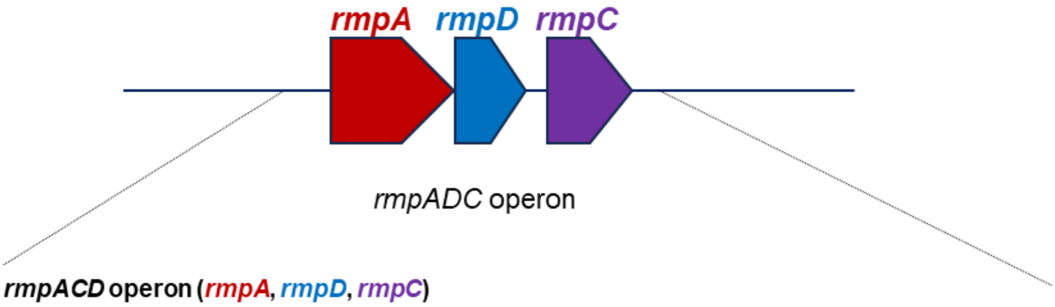

rmpACD **operon (*rmpA*, *rmpD*, *rmpC*)**

TATTCCGTTG GTAAGAATTT TCAAATAAGT CCTAAACTTC GCCCTCCCCA CACATCCTTA ATCATGAATT ATTAAGCAAA CTGACAAAAC AAGGGGGGGG
CGGAGTTAAA GAAAAGATCA ATTAATTGCA AACACGCAAA GGACAAGAAA TGACTTTTTA TTGTTATTGA TTGAATTTTT ATTCATTAAA AATTGTAACA
AACGACGTTC AAGAGAAATG ATTCTCTGTT GTTTTTTTTT TAAATGTATA TAAGATATTT TTGATTGATA TTGATGGATC AAAGTTACTG TTTATATGGA
AAAATATATT TACTTTATAT GTAATAGAGA TATAAATATC ATATTGACTG ATGATTATTT TTTTTATTAT GGTCTAAAGC AGTTAACTGG ACTACCTCTG
TTTCATATTA CATTTGAAGG AGTAATTAAT AAATCAATAG TTATTAAGCA CAAAAAAAAC ATAAGAGTAT TGGTTGACTG CAGGATTTTT TATTCAGGAA
AATGGAGAGG GTACAAAATG TTAAGGAAAT CATTAAATAT GATAAGCCAA TGGATATGGC TTGATGTTTC GGGGGGGGGG CGGTTTTACC CTAAAGGGTG
TGATTATGAC ATCTATGTTA ACATGCAAGG AAATGTAAAA AAAAATATTG AAAAACTATA TTTTGCATTC TTAAAGAAAA ATGTTAGCCG AATTGTAAAC
CATTATCCAC GGCTAACAAA AAAAGAACAA ATAGTACTGC AATGCTTACT GAAAAGTAAG GGGGGGGATG AAATAAAAAA TCAGCTAAAA ATTGAAGAAA
AAACACTATC ATGCTACCGA AGCAAGATAA CAAGAAAATT TGGCTGTAAA GGGTACATAA GGTTTATGTA TCTTTACAGT CTTAATAAAG AAATGGTTGA
TGAAAGATGT CTCATGCTAG GTATTTAGAA AAAAAGGGGA GGAGGGGGTG AAAGCACTCT TTCATTTATT TATTTTTTTA TTTCTGTTTT ATATATCAGT
TTATTGTTTT TATTCATATG TGTCAGATAG AAGAAGAATA AAAAAAATAT TCCGCTCGCA CAGAACATTG ATAAATAGAC GAAAAAAATC AAACTTAATA
AAATATATCA CATTCAATAA ATGATATCAG CGCGGTATAT AACTCACGAC AATTTTAGTT GGCAGATAAG TATGCATATT GCATGCGCCA TTTATGTATA
AAACACCTAT CAAAGATCTC AAAAAATAGG GTCACTATAC ATTATAATGT ATCTCCAGCA AATGAGAATA TATAAAGAAA AAAGGATTAA GGTAAGAAAA
ATAATGAGAT TGTGTGTCGT TACAGAAAAC ACTTATTTCT TTGCCGGCAT GGAATATATT TTCGCAGAGG TTCAATGCTG TCTTTGCAGA ATATCGAGTT
GTGATGTATA TGCATGCACT CCTGATTCAA ACGCAATATT TTTATTGGAT GGTGTGAATC ACAAAATTTC GATAAAAGAG TATAGCTATC TAAAAAAAAT
AGGCTCACCT GTTTTTTTTA TTCTGAACAC AAACTGTAAA GTTAATTCCA CCCTCATCGG AATTAACATC ATTGACGCAC ACGAGGCTAT CCTTATTCTA
AAGGATATCA TGCTTTCTCT ATTTAATGGG GGGAAACAGC TTGATTATAA ACCAATAAAT TTGACAAAGA AGGAATCTTT TGTTCTTAGA TTATATATAG
ATGGGTTATC ATTAACACAG ATAAGTGAAA AAATAGCCAT TAGAAAAAAA ACCCTTTTAA CTCACACACG GAATATTCTT AATAAGACCG GTGTAAAACA
CCACAATCAC CTTAATATTT TAAAAAACAT CCTTGGCATC CACTTAGCTT ATTAAAGATG GTATAACTTT TGGTGTCCAC AGATTTGTGC GTTTAAAATC
ATATACATTA TTAATGAAGG AGAATGAATG CGTAGAATTA CTATATTTAT ATTATTCCTG TTAGTTGCTG TAACTTGGGG AACCACCTGG ATAGCTATGA
AGATTGCTCT TGAAAGTATC CCTCCAGTCT TTGCTACCGG GATGAGATTT TTATTTTCCG CTCCATTATT AATCATTATC GCATGGGTAA AAAAAATACC
TCCGCTCGCA CAGAACATTG ATAAATAGAC GAAAAAAATC AAACTTAATA AAATATATCA CATTCAATAA ATGATATCAG CGCGGTATAT AACTCACGAC
AATTTAGTT GGCAGATAAG TATGCATATT GCATGCGCCA

Red character; *rmpA* ORF
Blue character; *rmpD* ORF
Purple character; *rmpC* ORF

Supplementary Figure S1. Schematic diagram of the capsular synthesis gene cluster (*cps* locus) (A) and *rmpADC* operon (B) on the *K. pneumoniae* chromosome. (A) *K. pneumoniae* encodes 20 chromosomal genes involved in capsular polysaccharide synthesis (cps). The *cps* locus comprises three operons, represented by *galF*, *wzi*, and *manC*. (B) Nucleotide sequence of the *rmpADC* operon obtained from the genome of *K. pneumoniae* ATCC43816. The *rmpA* ORF is shown in red, the *rmpD* in

Supplementary Figure S2

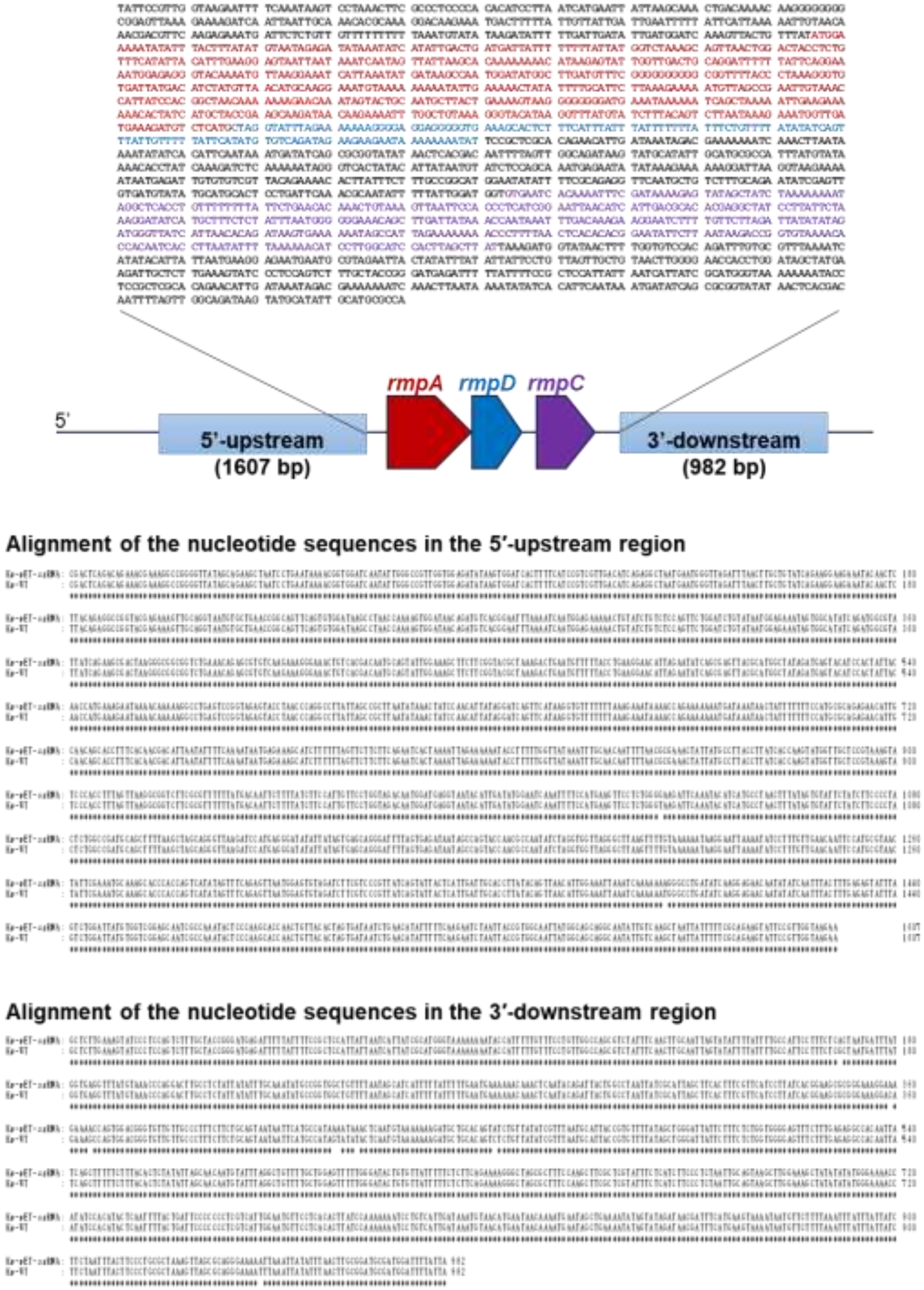

Supplementary Figure S2. Alignment of nucleotide sequences in the 5'- upstream and 3′ downstream regions of *rmpADC* in Kp-WT and Kp-pET-sgRNA strains.
